# Dynamic Regulation of ARGONAUTE4 within Multiple Nuclear Bodies in Arabidopsis thaliana

**DOI:** 10.1371/journal.pgen.0040027

**Published:** 2008-02-08

**Authors:** Carey F Li, Ian R Henderson, Liang Song, Nina Fedoroff, Thierry Lagrange, Steven E Jacobsen

**Affiliations:** 1 Molecular Biology Institute, University of California Los Angeles, Los Angeles, California, United States of America; 2 Department of Molecular, Cell and Developmental Biology, University of California Los Angeles, Los Angeles, California, United States of America; 3 Biology Department and Huck Institute of the Life Sciences, Pennsylvania State University, University Park, Pennsylvania, United States of America; 4 Laboratoire Génome et Développement de Plantes (LGDP), UMR 5096, Université de Perpignan, Perpignan Cedex, France; 5 Howard Hughes Medical Institute, University of California, Los Angeles, California, United States of America; Massachusetts General Hospital, United States of America

## Abstract

DNA methylation directed by 24-nucleotide small RNAs involves the small RNA-binding protein ARGONAUTE4 (AGO4), and it was previously shown that AGO4 localizes to nucleolus-adjacent Cajal bodies, sites of snRNP complex maturation. Here we demonstrate that AGO4 also localizes to a second class of nuclear bodies, called AB-bodies, which are found immediately adjacent to condensed 45S ribosomal DNA (rDNA) sequences. AB-bodies also contain other proteins involved in RNA-directed DNA methylation including NRPD1b (a subunit of the RNA Polymerase IV complex, RNA PolIV), NRPD2 (a second subunit of this complex), and the DNA methyltransferase DRM2. These two classes of AGO4 bodies are structurally independent—disruption of one class does not affect the other—suggesting a dynamic regulation of AGO4 within two distinct nuclear compartments in *Arabidopsis*. Abolishing Cajal body formation in a *coilin* mutant reduced overall AGO4 protein levels, and *coilin dicer-like3* double mutants showed a small decrease in DNA methylation beyond that seen in *dicer-like3* single mutants, suggesting that Cajal bodies are required for a fully functioning DNA methylation system in *Arabidopsis*.

## Author Summary

Argonautes are components of the RNA interference (RNAi) pathway that bind small interfering RNAs (siRNAs) of 21–24 nucleotide length. In the flowering plant Arabidopsis thaliana, ARGONAUTE4 (AGO4) is involved in gene silencing at the transcriptional level in a process called RNA-directed DNA methylation (RdDM), during which siRNAs cause transcriptional gene repression at complementary sequences. Previously, we found that AGO4 localized to nucleolus-adjacent Cajal bodies, sites of snRNP complex assembly. In this study, we show the existence of a second class of AGO4 nuclear foci, which we call the “AB-bodies,” that is distinct from the Cajal body and is immediately adjacent to the condensed 45S ribosomal DNA (rDNA) loci. In addition to AGO4, AB-bodies also contained NRPD1b and NRPD2 (subunits of the plant-specific RNA polymerase IV complex) and the DNA methyltransferase DRM2. The two different classes of AGO4 nuclear foci are structurally distinct, since the loss of one class did not affect the other. Losing Cajal bodies resulted in the enhancement of the DNA methylation defects seen in the RNA silencing mutant *dicer-like3*, suggesting Cajal bodies are required for a fully functioning RdDM pathway leading to gene silencing.

## Introduction

RNA-directed DNA methylation (RdDM) is a phenomenon discovered in plants in which small RNAs cause DNA methylation and transcriptional gene silencing at complementary sequences in the genome [1–3]. Frequent targets of RdDM include regions which contain direct and/or inverted repeats such as those present in transposable elements, some intergenic regions, and a small percentage of promoters at endogenous genes [4]. Numerous studies have linked components of the RNA interference (RNAi) pathway to RdDM [5]. In RNAi, double stranded RNA substrates are cleaved by the endonuclease Dicer to form small interfering RNAs (siRNAs) which are then bound by an Argonaute protein [6]. Using the siRNA as a guide, Argonautes can direct gene silencing through transcriptional or post-transcriptional mechanisms [7,8].

In *Arabidopsis*, genes involved in the production of siRNAs associated with DNA methylation include *NRPD1a*, *RNA-DEPENDENT RNA POLYMERASE2* (*RDR2*), and *DICER-LIKE3* (*DCL3*) [9–12]. Downstream genes which carry out the transcriptional silencing function directed by the siRNAs include *ARGONAUTE4* (*AGO4*) and *NRPD1b* [13–15]. *NRPD1a* and *NRPD1b* encode isoforms of the largest subunit of the plant-specific RNA polymerase IV complex (RNA PolIV), but depending on which subunit is present in the complex, RNA PolIV can function upstream in siRNA biosynthesis (NRPD1a) or downstream in causing DNA methylation (NRPD1b) [9,10,14,15]. The final step of the RdDM pathway is performed by DOMAINS REARRANGED METHYLTRANSFERASE2 (DRM2), a de novo DNA methyltransferase that is required for the establishment of DNA methylation and for the maintenance of DNA methylation in asymmetric CHH (where H=A, T, or C) and CHG sequence contexts [12,16]. Mutations in these RdDM genes result in decreased transcriptional silencing of certain repetitive loci.

Past studies investigating the subcellular distribution of nuclear proteins have uncovered a spatial partitioning of functions within the nucleus [17]. Distinct nuclear compartments or bodies with functions such as splicing, RNA metabolism, or complex formation have been characterized [18]. One such nuclear compartment is the nucleolus-adjacent Cajal body, at which small nuclear RNAs (snRNAs) are modified and assembled into small nuclear ribonucleoprotein (snRNP) complexes that are involved in splicing in the nucleoplasm [19–21]. Localization studies in *Arabidopsis* revealed that several RdDM components also formed nuclear bodies which can be found either adjacent to or within the nucleolus [21,22]. Proteins which formed such discrete nuclear bodies include RDR2, DCL3, AGO4, and NRPD1b. More recently, Dicing bodies (D-bodies), which contain microRNA processing factors DCL1 and HYL1, were also discovered in plant nuclei [23,24]. These D-bodies are present in the nucleoplasm but are not associated exclusively with the nucleolus, unlike RDR2, DCL3, AGO4, and NRPD1b which have strong associations with the nucleolus.

In the case of AGO4, multiple AGO4 foci were often observed in a single nucleus, and these foci could be colocalized with either NRPD1b or the Cajal body [21]. Because NRPD1b was always found to be colocalized with AGO4, and because AGO4 was found to be localized to Cajal bodies in the vast majority of cells (95%), our previous interpretation was that AGO4, NRPD1b, and possibly other factors involved in RdDM were all localized to Cajal bodies [21]. However, in the present study we demonstrate the existence of two distinct classes of AGO4 bodies: a large population that colocalizes with Cajal bodies, and a second smaller population that colocalizes with NRPD1b (which we call “AB-bodies”). AB-bodies also contained two other RdDM components, namely NRPD2 and the DNA methyltransferase DRM2, and this body was found to be adjacent to the 45S ribosomal DNA (rDNA) loci, suggesting that the AB-body might be a site of active RNA-directed DNA methylation. AB-bodies and Cajal bodies showed distinct genetic requirements because a *coilin* mutation that disrupts Cajal body formation eliminated AGO4 at Cajal bodies but not at AB-bodies, and mutation of *NRPD1b* eliminated AGO4 at AB-bodies but not at Cajal bodies. These results suggest that AGO4 localization is regulated in a complex fashion involving two structurally distinct nuclear compartments. Finally, we found that disruption of Cajal body formation decreased the overall protein level of AGO4 and enhanced the weak effect of a *dcl3* mutation on DNA methylation at two repetitive loci (*MEA-ISR* and 5S ribosomal DNA), suggesting that Cajal bodies are required for a fully functioning RdDM system.

## Results

### Two Distinct Classes of AGO4 Nuclear Bodies in *Arabidopsis* Nuclei

**Figure 1:**
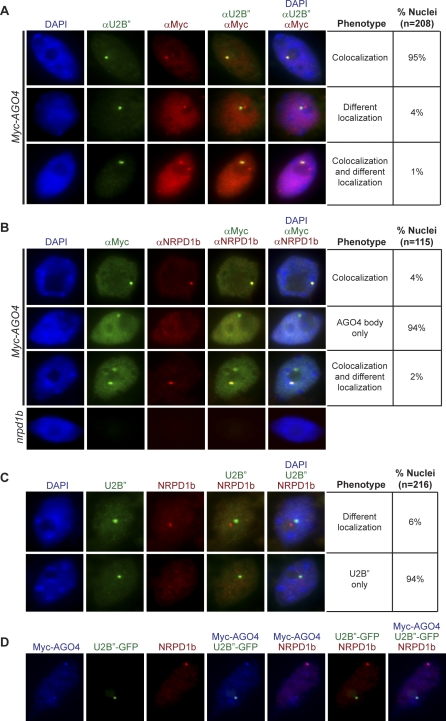
AGO4 Localizes to Two Distinct Nuclear Bodies (A) Fluorescent microscopy analysis of Myc-AGO4 and Cajal body localization in nuclei isolated from Myc-AGO4 seedlings. A monoclonal antibody to endogenous U2B′′ was used to detect the Cajal body. Three different AGO4 localization patterns relative to U2B′′ are shown. Nuclei containing observable AGO4 nuclear foci were examined. (B) Localization of Myc-AGO4 and NRPD1b in Myc-AGO4 nuclei. A polyclonal antibody was used to detect endogenous NRPD1b. Three representative nuclei are shown. Nuclei containing observable AGO4 nuclear foci were examined. (C) Localization of U2B′′ relative to NRPD1b within wild type Ler nuclei. (D) Three color fluorescent microscopy analysis of Myc-AGO4, U2B′′-GFP, and NRPD1b localization within the same nucleus. One representative nucleus containing two different AGO4 bodies is shown: colocalization with NRPD1b (top of the nucleus) versus colocalization with the Cajal body (bottom of the nucleus). All AGO4 foci colocalized with either NRPD1b or Cajal body (*n* = 156).

Utilizing a plant line containing Myc-tagged genomic AGO4 that is under the control of its endogenous promoter, we previously found that AGO4 shows very good colocalization with Cajal body markers, with colocalization seen in roughly 95% of the nuclei [21]. Here we undertook an examination of a large population of nuclei in order to study the small fraction of nuclei in which AGO4 and Cajal body markers did not colocalize. Within about 200 nuclei isolated from Myc-AGO4 seedlings, we confirmed that a majority of nuclei contained AGO4 colocalizing with U2B′′, a marker for the Cajal body (Figure 1A) [21,25,26]. However, we also observed a small population (4%) of nuclei that contained AGO4 foci that did not colocalize with U2B′′, and an even smaller population (1%) of nuclei which displayed both types of AGO4 localization (one colocalized with U2B′′ and one not colocalized with U2B′′). We confirmed this result by examining AGO4 localization relative to SmD3 and SmB, which are two proteins also enriched at the Cajal body [21,27]. Non-colocalization between AGO4 and SmD3 or SmB was observed in a small population of nuclei (Figure S1; Table S1), again suggesting that AGO4 forms nuclear foci distinct from the Cajal body.

An examination of AGO4 relative to NRPD1b localization within nuclei was also performed to determine if there are AGO4 foci that are separate from NRPD1b. As observed previously (see Table S1 in [21]), NRPD1b colocalized with AGO4 in a small percentage of cells, while the remainder of the nuclei contained only AGO4 foci and no NRPD1b (Figure 1B). Also as previously observed, when analyzing those nuclei in which clear NRPD1b foci were present, those NRPD1b foci always colocalized with AGO4. However, we observed a small population of nuclei (2% in the experiment shown in Figure 1B) in which, in addition to the NRPD1b and AGO4 containing bodies, a second focus of AGO4 staining was found that did not colocalize with NRPD1b.

These results suggest the possibility of multiple different AGO4 bodies, and suggest that NRPD1b might not localize to Cajal bodies as was previously assumed [21]. To test this hypothesis, we examined U2B′′ localization relative to NRPD1b in nuclei and found that these two proteins were indeed localized to separate foci adjacent to the nucleolus (Figure 1C). Utilizing three fluorescent colors to look at Myc-AGO4, GFP-U2B′′, and NRPD1b simultaneously, we confirmed that multiple AGO4 foci within the nucleus either colocalized with NRPD1b or U2B′′, but we never observed all three proteins colocalizing together (Figure 1D). Further characterization of the two different AGO4 bodies by fluorescence measurements using confocal microscopy showed that AGO4 immunostaining at the NRPD1b body was generally more intense and punctate, while AGO4 immunostaining at the Cajal body was usually less intense and more diffuse (Figure S2). We also tested whether any of the AGO4 foci corresponded to Dicing bodies which contain HYL1 and DCL1 [23,24]. By co-immunofluorescence analysis, GFP-HYL1 did not colocalize with AGO4 or NPRD1b, suggesting none of the different AGO4 bodies are Dicing bodies (Figure S3A and S3B; Table S2). GFP-HYL1 also did not colocalize with U2B′′, consistent with published results which showed Dicing bodies are not Cajal bodies (Figure S3B; Table S2) [23,24]. These results suggest there are two classes of AGO4 nuclear bodies: a first class that colocalizes with the Cajal body in a majority of nuclei, and a second class that is with NRPD1b in a smaller population of cells (called “AB-bodies” for “AGO4/NRPD1b bodies”).

### Cajal Body Integrity Affects AGO4 Localization and Is Required for a Fully Functioning RdDM System

**Figure 2:**
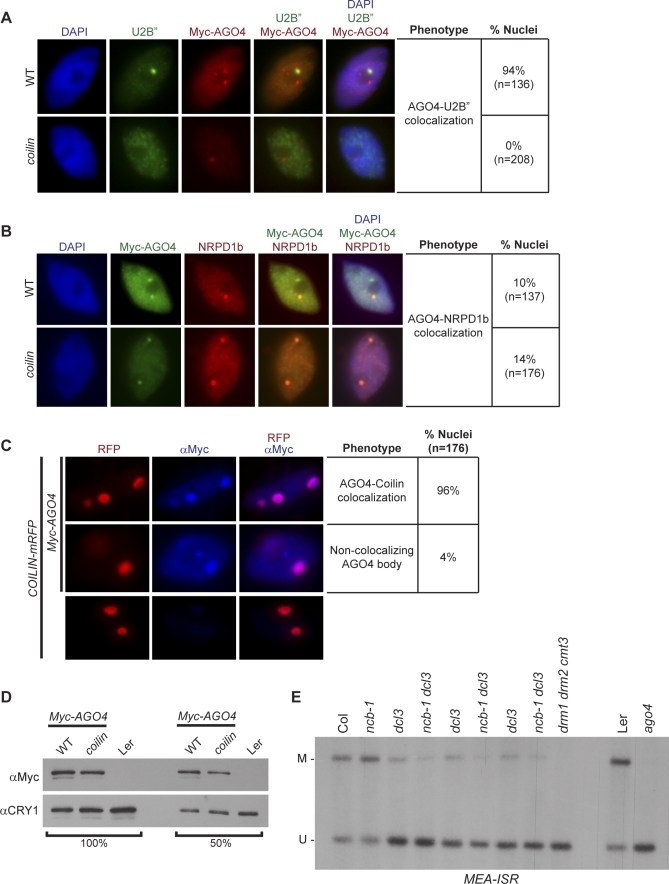
Effects of the Cajal Body on AGO4 Localization and DNA Methylation (A,B) Immunofluorescence analysis of Myc-AGO4 localization in wild type nuclei or a *coilin* mutant (SALK 148589) is shown relative to (A) U2B′′ or (B) NRPD1b. (C) Myc-AGO4 nuclear distribution in a plant line overexpressing *COILIN-mRFP* and containing enlarged Cajal bodies. (D) Semi-quantitative western blot analysis of Myc-AGO4 protein. Total protein amounts of 17 μg (labeled as 100%) or 8.5 μg (labeled as 50%) were loaded per sample. An antibody to c-Myc (Upstate) was used to detect Myc-AGO4. The endogenous photoreceptor CRY1 was used as a loading control [47]. (E) Southern blot analysis examining DNA methylation at the *MEA-ISR* locus. Genomic DNA was digested with methyl-sensitive enzyme *Msp*I (^m^CCGG). Three independent biological replicates of *dcl3* and *ncb-1 dcl3* were examined. The band indicating methylated DNA (undigested) is labeled as “M”, while the band indicating unmethylated DNA (digested) is labeled as “U”.

The presence of the two classes of AGO4 bodies led us to ask whether the Cajal body and AB-body might be dependent on one another for stability. To investigate this, Myc-AGO4 and NRPD1b localization were examined in a *coilin* mutant that was unable to form visible Cajal bodies (Figure S4). Mutations in *COILIN* were shown to completely disrupt Cajal body formation, and interestingly, this disruption did not cause any obvious morphological defect in plant development [28]. We found that in the *coilin* mutant background, only a small fraction of nuclei (14%) contained AGO4 foci, and these AGO4 foci all colocalized with NRPD1b (Figure 2A and 2B; Table S3). This indicates that AGO4 is no longer able to form nuclear foci outside of the AB-bodies in the absence of the Cajal body. This finding that only AB-bodies were observed in the *coilin* mutant suggests that the formation of the AB-body is not affected by Cajal body integrity or by the ability of AGO4 to localize to the Cajal body. Interestingly, in a plant line that overexpressed *COILIN-mRFP* and contained enlarged Cajal bodies [28], AGO4 enrichment at the Cajal body also expanded to the same size as the enlarged Cajal body, while no major effect on the AB-body was observed (Figure 2C).

In addition to the effects on AGO4 localization, we also observed less intense staining of Myc-AGO4 both within nuclear foci and throughout the nucleoplasm in the *coilin* mutant (Figure 2A and 2B). This decrease in AGO4 detection is reminiscent of the effects of *rdr2*, *dcl3*, and *nrpd1a* on AGO4, where a mutation in any of these genes decreased overall protein levels of AGO4 [21]. To confirm that the observed decrease in AGO4 staining intensity in *coilin* nuclei is also due to lower AGO4 protein levels, a semi-quantitative western blot analysis was used to examine Myc-AGO4 levels in *coilin* or a wild type sibling. Consistent with the immunofluorescence analysis, the overall protein level of Myc-AGO4 was slightly decreased in *coilin* when compared to wild type (Figure 2D). This finding suggests that the Cajal body is required for maintaining full levels of the AGO4 protein.

Next we tested whether proper Cajal body formation was required for AGO4 dependent RNA-directed DNA methylation. Thus, we examined DNA methylation and siRNA production in the *coilin* mutant and *COILIN-mRFP* overexpression line. No obvious change in DNA methylation was observed at the 5S rDNA loci or at the euchromatic locus *MEA-ISR* in the overexpression line, in *coilin* SALK mutant lines that we have isolated, or in the previously described Cajal body mutant lines (*ncb-1*, *ncb-2*, and *ncb-4*) (Figure S5A and S5B) [28]. Similarly, there was no effect on 45S and 5S siRNAs levels when the Cajal body was absent or expanded (Figure S5C). This lack of a general effect on gene silencing pathways is consistent with *coilin* mutants also lacking observable developmental defects even though Cajal bodies are a major site of snRNA modification and snRNP assembly [28].

Because the effect of *coilin* on AGO4 levels was only slight, we reasoned that we may only see an effect of losing the Cajal body on DNA methylation in a sensitized background in which the DNA methylation pathway has already been partially affected. Thus, we crossed a *coilin* mutant (*ncb-1*) to *dcl3*. The *dcl3* mutant was shown previously to have weaker effects than *rdr2*, *nrpd1a*, or *ago4* on DNA methylation [11,12], which in part could be due to DCL redundancy [21,29–31]. At *MEA-ISR*, the *ncb-1 dcl3* double mutant contained further decreased DNA methylation levels beyond that seen in *dcl3* alone (Figure 2E). This enhancement of a weak *dcl3* phenotype was slight but consistently reproducible using independent biological replicates (Figure 2E). Similar to *MEA-ISR*, we also observed a slight decrease in DNA methylation levels at the 5S rDNA loci in the *ncb-1 dcl3* double mutant relative to *dcl3* alone (Figure S5D). Hence, Cajal body integrity appears to have some role in RdDM efficiency, possibly through an effect on AGO4 protein levels.

### Stability of the AB-Body in RdDM Mutants

**Figure 3:**
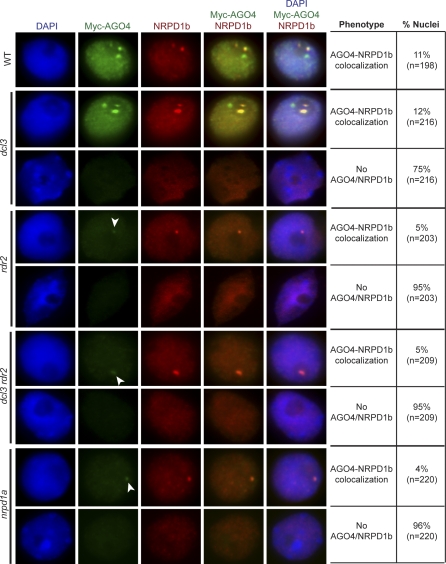
Presence of the AB-Body in Upstream RdDM Mutants Immunolocalization analysis of Myc-AGO4 and NRPD1b in wild type, *dcl3*, *rdr2*, *dcl3 rdr2*, or *nrpd1a* nuclei. White arrows indicate the presence of a faintly staining Myc-AGO4 body that colocalized with NRPD1b. Two populations of nuclei are shown for each mutant: nuclei with and nuclei without AB-bodies.

We investigated the factors that are important for the stability of the AB-body by examining the genetic requirements for its formation. In the upstream RdDM mutants *dcl3*, *rdr2*, *nrpd1a*, and *dcl3 rdr2*, NRPD1b nuclear bodies were still seen in a small percentage of nuclei similar to the percentage seen in wild type, despite slightly decreased fluorescence intensity (Figure 3; Table S4). Thus, NRPD1b does not require NRPD1a, RDR2, or DCL3 for localization to nuclear bodies.

We also examined AGO4 localization to the AB-body in the upstream mutants. As seen previously, AGO4 nuclear levels were severely decreased in a portion of *dcl3* nuclei and in all of the *rdr2*, *dcl3 rdr2*, and *nrpd1a* nuclei (Figure 3; Table S4) [21]. In the case of *dcl3*, only about one fourth of total nuclei from *dcl3* retained normal AGO4 levels, while the remaining majority of nuclei contained reduced AGO4 signal (Figure 3; Table S4). The *dcl3* mutant is known to have a weaker effect than *rdr2*, *nrpd1a*, or *ago4* on DNA methylation [11,12]*.* This lack of a stronger effect may be a result of the loss of AGO4 protein in only a fraction of *dcl3* nuclei, in addition to redundancies in DCL function [29–31]. The remaining *dcl3* cells that retain normal AGO4 levels would presumably allow some activity of RdDM to occur, giving an intermediate phenotype that falls between wild type and stronger mutants such as *rdr2*, *nrpd1a*, and *ago4*. Thus, some of the DNA methylation defects seen in *dcl3* may be an indirect effect of having less AGO4 protein, in addition to the more direct effect of losing siRNAs that are normally produced by DCL3 [11,29].

Within the population of *dcl3* nuclei that contained decreased AGO4 levels, no AGO4 or NRPD1b foci were observed (Figure 3; Table S4). In contrast, *dcl3* nuclei which had normal AGO4 levels contained both AGO4/Cajal bodies and AB-bodies (Figure 3; Table S4). It remains unclear what cell types within the *dcl3* mutant contain normal AGO4 levels, and why AGO4 protein should be stabilized in these specific cells. One explanation would be that another DCL is expressed in these cells, and in the absence of DCL3, replaces the function of DCL3 in stabilizing AGO4 protein and localization [29–31]. Alternatively, the *dcl3* mutation might result in a stochastic loss of AGO4 protein within certain nuclei that may or may not be dependent on cell type (discussed in [21]).

In the *rdr2*, *dcl3 rdr2*, and *nrpd1a* mutants, despite the low overall fluorescence levels, we observed a faint AGO4 body that colocalized with NRPD1b in all nuclei that contained NRPD1b bodies (Figure 3; Table S4). This is in contrast to *dcl3*, where AB-bodies were found in *dcl3* nuclei containing normal AGO4 levels but not in *dcl3* nuclei that lost the majority of AGO4 protein. This would suggest that the population of nuclei that contain AB-bodies in the *rdr2*, *dcl3 rdr2*, and *nrpd1a* mutant backgrounds are among those that also show normal AGO4 levels in a *dcl3* mutant (see Discussion). In summary, these results suggest that localization of both NRPD1b and AGO4 to AB-bodies still occurs in the *nrpd1a*, *rdr2*, or *dcl3* mutants.

**Figure 4:**
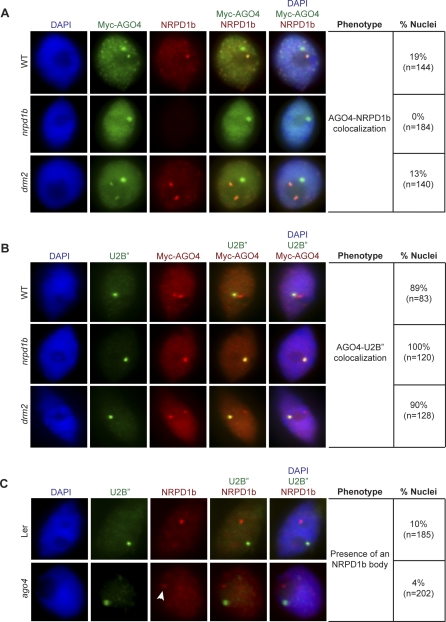
Effects of Downstream RdDM Mutations on the AB-Body (A,B) Myc-AGO4 nuclear localization in wild type, *nrpd1b*, or *drm2* mutant nuclei is shown relative to the immunostaining of (A) NRPD1b or (B) U2B′′. (C) Immunolocalization of NRPD1b and U2B′′ in wild type (Ler) or an *ago4* mutant. The white arrow indicates a less intense NRPD1b body that still remained in *ago4*.

To examine the effects of downstream mutations in the formation of the two classes of AGO4 bodies, we investigated whether AGO4/Cajal bodies or AB-bodies were present in *nrpd1b*, *ago4* and *drm2* mutant nuclei. In *nrpd1b*, a majority of cells still contained AGO4 foci (Table S4), consistent with previous results [21]. However, all of the remaining AGO4 foci present in *nrpd1b* colocalized with U2B′′, indicating that AGO4 is not able to form nuclear foci outside of the Cajal body in this mutant (Figure 4A and 4B; Table S4). Because only AGO4/Cajal bodies remain in the *nrpd1b* mutant, this suggests that the formation of the AB-body is dependent on NRPD1b while the localization of AGO4 to the Cajal body is not. In the *ago4* mutant however, NRPD1b bodies were still observable, although the overall fluorescence appeared to be slightly weaker than wild type (Figure 4C; Table S5). In the *drm2* mutant, we observed the presence of NRPD1b and both classes of AGO4 bodies, indicating that both AB-bodies and AGO4/Cajal bodies are unaffected in this mutant (Figure 4A and 4B; Table S4). These results suggest NRPD1b is upstream of both AGO4 and DRM2 in AB-body formation, and NRPD1b localization to nuclear bodies is mostly independent of AGO4.

### Localization of NRPD2 to AB-Bodies

**Figure 5:**
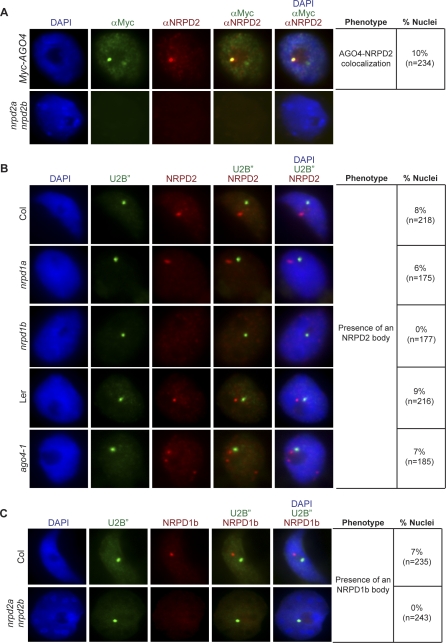
Localization of NRPD2 Relative to the AB-Body (A) Immunofluorescence analysis of NRPD2 localization relative to AGO4 in Myc-AGO4 nuclei. A polyclonal antibody was used to detect endogenous NRPD2 protein. (B) Localization of NRPD2 relative to U2B′′ in wild type, *nrpd1a*, *nrpd1b*, or *ago4* mutant nuclei. Ler is the wild type control for *ago4* within the same ecotype, and Col is the wild type control for *nrpd1a* and *nrpd1b*. (C) NRPD1b nuclear body formation in wild type versus *nrpd2a nrpd2b*. Nuclei were co-immunostained with an antibody to U2B′′.

*NRPD2* encodes the second largest subunit of RNA PolIV complexes containing NRPD1a or NRPD1b [9,14,15]. NRPD2 was shown to interact with NRPD1a and NRPD1b in vivo, and was observed to be colocalized with NRPD1a and NRPD1b in small speckles outside of the nucleolus [22]. To determine if NRPD2 is also present at the AB-body, the localization of NRPD2 within nuclei was examined using an antibody which detects endogenous NRPD2 [22]. By immunofluorescence analysis, we observed NRPD2 localized to a nucleolus-adjacent body that colocalized with AGO4 in a small population of nuclei (Figure 5A and S6). However, unlike AGO4, these NRPD2 nuclear bodies did not colocalize with U2B′′, indicating NRPD2 is not enriched at the Cajal body (Figure 5B; Table S6). Since NRPD2 is at an AGO4 focus that is not a Cajal body, this strongly suggests that NRPD2 is also targeted to the AB-body. Although we could not directly test colocalization of NRPD2 with NRPD1b since antibodies to NRPD2 and NRPD1b are both rabbit polyclonal, our observed NRPD2 body is most likely the AB-body given the finding that NRPD2 interacts with NRPD1b in vivo [22], and NRPD2 also colocalized with a fourth protein present at the AB-body, DRM2 (see below).

To determine whether NRPD2 displays the same genetic requirements as AGO4 and NRPD1b for localization to the AB-body, the ability of NRPD2 to form nuclear bodies was examined in *nrpd1a, rdr2*, *ago4*, and *nrpd1b* mutants. Similar to NRPD1b, NRPD2 nuclear bodies were still visible at wild type fluorescence levels in *nrpd1a* (Figure 5B; Table S6) and *rdr2* (not shown), and still present with slightly decreased intensity levels in *ago4* (Figure 5B; Table S6). However, in the *nrpd1b* mutant, no obvious nuclear bodies were seen for NRPD2 (Figure 5B; Table S6). We performed the reciprocal experiment and found no obvious NRPD1b nuclear body in the *nrpd2* mutant (Figure 5C; Table S5). This lack of NRPD1b or NRPD2 bodies in *nrpd2* or *nrpd1b*, respectively, is likely in part due to the fact that NRPD1b and NRPD2 depend upon each other for protein stability [14]. However, it is also possible that the absence of NRPD1b or NRPD2 bodies is due to a defect in targeting to the AB-body in the *nrpd2* or *nrpd1b* mutant background, respectively. These results suggest NRPD1b and NRPD2 show similar requirements with regards to localization to AB-bodies.

### AB-Bodies Are Always Adjacent to Condensed 45S rDNA Loci

**Figure 6:**
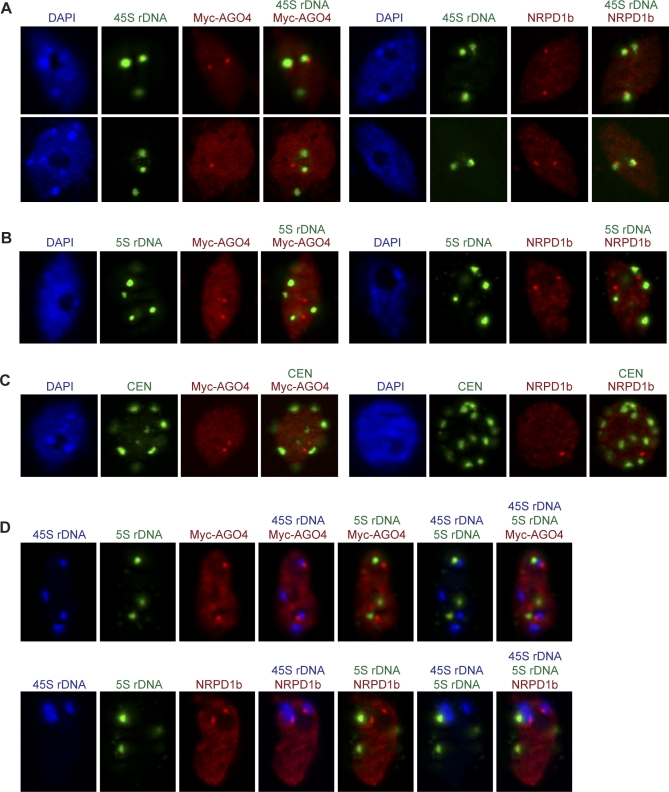
Localization of AB-Bodies to the Immediate Proximity of the 45S rDNA Loci (A) DNA FISH analysis of the condensed 45S rDNA loci (NORs) relative to Myc-AGO4 (left) or NRPD1b (right) localization in Myc-AGO4 or Ler nuclei, respectively. (B,C) Localization of AGO4 or NRPD1b is shown relative to (B) 5S rDNA loci or (C) centromeric repeats (*CEN*). (D) Dual probe DNA FISH analysis showing 45S and 5S rDNA loci relative to Myc-AGO4 (top panel) or NRPD1b (bottom panel). The 45S probe was labeled with biotin and the 5S probe was labeled with digoxigenin (DIG). NOR4 is observed as a 45S rDNA signal with an adjacent 5S rDNA signal, while NOR2 shows only 45S rDNA signal with no adjacent 5S rDNA signal.

To further characterize the subnuclear localization of AB-bodies, DNA fluorescence in situ hybridization (FISH) in combination with protein immunofluorescence analysis was performed to visualize the location of major repetitive genomic regions (45S rDNA, 5S rDNA, and centromeric repeats) relative to the AB-body. As seen previously, a significant population of Myc-AGO4 nuclei showed AGO4 foci separate from the condensed 45S rDNA loci (or nucleolar organizer regions, NORs) (Figure 6A, bottom panel; Table S7) [21,32], since most of the nuclei show AGO4 at Cajal bodies. However, we also observed a population of AGO4 foci that were immediately adjacent to or slightly overlapping with the NORs (Figure 6A, top panel and Figure S7; Table S7). One AGO4 focus per NOR was frequently observed, although two or three AGO4 foci surrounding one NOR were also seen (Figure S8A).

To confirm that the AGO4 foci associated with the condensed 45S rDNA loci were AB-bodies and not Cajal bodies we performed DNA FISH together with NRPD1b immunolocalization. In all nuclei containing NRPD1b bodies, NRPD1b was always found adjacent to or overlapping with the NORs, indicating that the NOR-adjacent AGO4 foci are AB-bodies (Figure 6A; Table S7). Consistent with this result, we did not find the Cajal body, either marked by GFP-SmD3 or GFP-SmB fluorescence, to be closely associated with the NORs (Figure S8B; Table S7). In contrast to the NORs, AGO4 or NRPD1b foci were not found to be closely associated with the 5S rDNA loci or centromeric repeats (CEN) (Figure 6B, 6C, and S7; Table S7). These results suggest that the AB-body is specifically situated adjacent to the NORs.

Since the 45S rDNA repeats are present on both chromosome 2 (NOR2) and chromosome 4 (NOR4) [33], it is possible that AGO4/NRPD1b may be preferentially targeted to one NOR versus another. We examine this possibility by performing dual DNA FISH analysis looking at both condensed 45S and 5S rDNA loci simultaneously in addition to AGO4 or NRPD1b immunofluorescence. Since the 5S rDNA loci are present on chromosome 4 but not on chromosome 2 [34,35], NOR4 can be detected by the close proximity of the 45S signal to 5S signal, while NOR2 will only display 45S signal and no adjacent 5S signal [33]. Using this method, we found AB-bodies that were adjacent to both NOR2 (45S only) and NOR4 (45S with 5S) (Figure 6D; Table S8). This suggests that the AB-body is present at both NORs.

### The AB-Body Associates with DRM2

**Figure 7:**
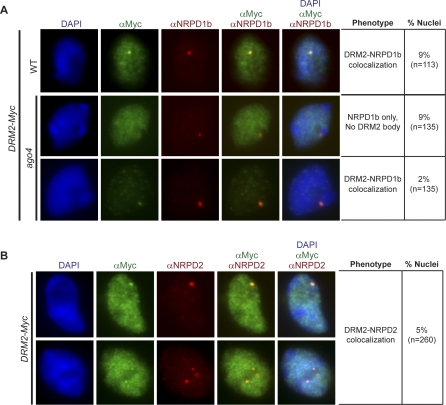
Localization of DRM2 with AB-odies (A) DRM2-Myc nuclear localization relative to NRPD1b in wild type or an *ago4* mutant. (B) Immunofluorescence analysis of the NRPD2 nuclear body relative to the DRM2 body.

The finding that both AGO4 and NRPD1b are associated with the NORs suggests that the AB-body might be a site of active transcriptional silencing at the 45S rDNA repeats. The 45S rDNA loci consist of both silent and active copies of the 45S rDNA gene [32]. It is possible that some of these active rDNA copies may undergo de novo silencing by the RdDM machinery in the form of the AB-body, especially since 45S rDNA silencing in *Arabidopsis* is known to involve DNA methylation at promoter sequences [36]. If this is the case, the de novo DNA methyltransferase DRM2 might also colocalize with the AB-body. To test this, we utilized a stable plant line expressing Myc-tagged genomic *DRM2* which is under the control of its endogenous promoter [21]. We examined the localization of DRM2 in nuclei which contained AB-bodies marked with NRPD1b or NRPD2. As previously observed, a majority of nuclei contained DRM-Myc localized diffusely throughout the nucleoplasm with less intense signal at chromocenters (Table S9) [21]. In a small population of nuclei where NRPD1b or NRPD2 bodies were present, DRM2 was also observed as a nucleolus-adjacent body that colocalized with NRPD1b or NRPD2 (Figure 7A and 7B; Table S9). In contrast, no DRM2 body was seen colocalized with U2B′′, indicating DRM2 is not enriched at the Cajal body (Figure S9) [21]. Interestingly, the *ago4* mutation caused a drastic reduction in the number of nuclei containing DRM2 bodies, suggesting that AGO4 has a role in targeting DRM2 to the AB-body (Figure 7A; Table S9). We also attempted to determine whether DRM2 localization is affected in *nrpd1b*. However, due to the speckle-like nucleoplasmic staining of DRM2 that is only slightly fainter than the DRM2 signal at the AB-body, and without a second marker (such as NRPD1b or NRPD2) to confirm localization, it is difficult to conclusively determine whether DRM2 is still able to form a nuclear body that colocalizes with the AB-body in *nrpd1b*. However it is unlikely that DRM2 would be targeted to the AB-body in the absence of NRPD1b, since AGO4 localization to the AB-body requires NRPD1b, and DRM2 localization to the AB-body requires AGO4. In sum, the presence of DRM2 at the AB-body and its dependence on AGO4 are consistent with the idea that the AB-bodies might represent active sites of gene silencing.

## Discussion

Our findings demonstrate the existence of two independent populations of AGO4 nuclear bodies that are structurally distinct. This is in contrast to the model that we proposed previously in which we assumed that all AGO4 bodies were Cajal bodies, including AGO4 bodies that colocalized with NRPD1b [21]. Examination of a large population of nuclei combined with direct testing of the localization of NRPD1b relative to the Cajal body demonstrated that our initiation interpretation was incorrect, and that the regulation of AGO4 distribution within the nucleus is more complex than first conceived. Our new data show that AGO4 localizes independently to the Cajal body or to the 45S rDNA-adjacent AB-body, and that AB-bodies are distinct from AGO4/Cajal bodies.

The majority of AGO4 foci within the nucleus represents the Cajal body, a nucleolus-adjacent compartment in which the maturation of snRNP complexes occurs. Our analysis with a *coilin* mutant showed that AGO4 levels were affected by the absence of Cajal bodies. This suggests that the Cajal body may play a role in stabilizing AGO4 protein, just as RdDM components RDR2, DCL3, and NRPD1a are also involved in AGO4 stability. Although no obvious DNA methylation defects were seen in the *coilin* mutants alone, the *ncb-1* mutation enhanced the DNA methylation phenotype of *dcl3* at *MEA-ISR* and the 5S rDNA loci. The *ncb-1 dcl3* double mutant had less DNA methylation than *dcl3* alone but did not decrease methylation to the levels seen in *ago4* or the triple DNA methyltransferase mutant *drm1 drm2 cmt3*. One explanation for the lack of a stronger enhancement by *ncb-1* is that the function of the Cajal body may still partially remain in the *coilin* mutant. It is possible that although visible Cajal body formation is disrupted, Cajal body function may still exist in smaller, less concentrated foci through the nucleus in *coilin* mutants. This is a likely possibility since Cajal body mutants display no obvious developmental or morphological defects, despite the fact that Cajal bodies are major centers for assembly of splicing complexes that are essential for cellular processes [28]. A similar situation exists in mammals where mice unable to form wild type Cajal bodies as a result of a deletion in coilin are also largely aphenotypic [37]. In turn, AGO4 function at the Cajal body also may be dispersed throughout the nucleus in the absence of a visible concentrated Cajal body. Despite the weak phenotype, our data showing the effects of *coilin* on AGO4 stability and on enhancing DNA methylation defects in the *dcl3* mutant suggest that the Cajal body plays some role in the efficiency of RdDM.

The smaller population of AGO4 foci, the AB-bodies, is present in nuclei at a frequency that varies from experiment to experiment, typically ranging from 4% to 20% of total nuclei. The AB-bodies are present in every major tissue examined, such as flowers, leaves, and whole seedlings (data not shown). This suggests that AB-bodies might be present in cell types that constitute a minority of cells within most major tissues of the plant.

In addition to AGO4 and NRPD1b, AB-bodies also contain RdDM factors NRPD2 and DRM2. The AB-body was always observed to be associated with the NORs by DNA FISH analysis, suggesting that AB-bodies might be sites of on-going 45S rDNA silencing, especially since DRM2 is also enriched at these particular nuclear bodies. Some of the active rDNA repeats may be specifically targeted for de novo silencing by the RdDM machinery as a mechanism to modulate or fine tune total cellular rDNA gene activity. This idea is consistent with earlier findings showing that a subset of the rDNA repeats in *Arabidopsis* is more highly DNA methylated and less occupied by RNA polymerase I, while another subset is less DNA methylated and more highly associated with RNA polymerase I [36]. Interestingly, FISH analysis showed that the AB-body was most often adjacent to the NORs instead of overlapping them. One explanation for the minimal overlap is that the AB-body might be present at less condensed or active 45S rDNA sequences that require gene silencing, and that these less condensed 45S rDNA sequences are difficult to visualize by DNA FISH. Euchromatic regions were previously shown to loop out of adjacent condensed DNA regions called chromocenters, which contain highly condensed 45S rDNA, 5S rDNA, and/or centromeric sequences [33]. It is thus possible that the AB-body is present at one of these loops, which would then situate the AB-body adjacent to the NORs.

The stability of the AB-body in upstream RdDM mutants sets AB-bodies apart from previously characterized nuclear bodies and indicates that AB-bodies are novel nucleolus-associated bodies [22]. Interestingly, while AB-bodies were detectable in *nrpd1a*, *rdr2*, and *dcl3 rdr2* nuclei that contained very little overall AGO4 protein, we found that in *dcl3*, nuclei containing decreased overall AGO4 levels did not show any AB-bodies, but *dcl3* nuclei with normal AGO4 levels did contain AB-bodies (Figure 3; Table S4). One possible explanation for this observation is that the specific cell types that normally form AB-bodies exist within the population of *dcl3* nuclei that still contain normal AGO4 levels, and not within the *dcl3* population containing reduced AGO4 levels. While the molecular basis for this correlation is unknown, it suggests a relationship between the particular cell population or cell type that contains AB-bodies and those able to express normal levels of AGO4 despite the *dcl3* mutation.

The fact that AB-bodies were still detected in *nrpd1a*, *rdr2*, and *dcl3* mutants suggests that an alternative pathway exists to target AGO4 and NRPD1b to 45S rDNA or adjacent regions. The finding that NRPD1a and RDR2 are not required for AB-body formation may indicate that the target sequence of the AB-body can produce double stranded RNA and siRNAs in a manner that does not require the activity of NRPD1a-containing RNA PolIV complexes or RDR2. In this way these loci would show the same genetic requirements as do a variety of inverted repeat loci in the *Arabidopsis* genome whose siRNA synthesis does not require NRPD1a or RDR2 [38].

The finding that AGO4 did not localized to AB-bodies in the *nrpd1b* mutant, but that NRPD1b could still localize to nuclear bodies in an *ago4* mutant, suggests a function of NRPD1b in stabilizing AGO4 at the 45S rDNA loci, possibly by NRPD1b interacting with AGO4 directly [21] and/or by generating a nascent RNA strand for AGO4 binding. Our data would further suggest that once stably localized at the target region by NRPD1b, AGO4 would then recruit DRM2 (directly or indirectly), since the localization of DRM2 to the AB-body depends on AGO4. In the future, it will be interesting to determine the precise nature of the sequences targeted by components of the AB-body, and the mechanisms and factors that recruit NRPD1b, NRPD2, AGO4 and DRM2 to 45S rDNA adjacent sequences.

## Materials and Methods

### Generation of epitope tagged lines.

Genomic *AGO4* was N-terminally tagged with four copies of c-Myc and expressed under the control of the endogenous *AGO4* promoter as described [21]. Genomic *DRM2* was C-terminally tagged with nine copies of c-Myc and expressed under its endogenous promoter as described [21]. *GFP-SmD3* and *GFP-SmB* plasmids, and *U2B′′-GFP* and *COILIN-mRFP* plant lines were gifts from Peter J. Shaw. Construction of *GFP-SmD3* and *GFP-SmB* plasmids were described previously [39]. *GFP-SmD3* and *GFP-SmB* were transformed into *Myc-AGO4* plants using *Agrobacterium* strain AGL1. Construction and generation of *U2B′′-GFP* and *COILIN-mRFP* lines were described previously [28]. The *U2B′′-GFP* or *COILIN-mRFP* line was crossed to *Myc-AGO4* plants to generate lines containing both epitopes. Construction of the *GFP-HYL1* plasmid was described previously [24]. *Myc-AGO4* plants were transformed with *GFP-HYL1* to produce transgenic plants containing both *Myc-AGO4* and *GFP-HYL1*. For mutant analysis, epitope tagged lines were crossed to *nrpd1a*, *dcl3 rdr2*, *ago4–1*, *nrpd1b*, or *drm2* and homozygosed for the single or double mutations.

### Loss-of-function mutants.

The RdDM mutants used in this study are the following: *nrpd1a* (SALK 143437, ABRC stock center), *dcl3–1* and *rdr2–1* [11]; *ago4–1* [13], *drm2* [16], *nrpd1b-1* [14], and *nrpd2a-2 nrpd2b-1* [15]. The DNA methyltransferase triple mutant *drm1 drm2 cmt3* was isolated as described [40]. The *coilin* mutants SALK 148589, SALK 148630, SALK 010395, and SALK 083448 were obtained from the ABRC stock center. Published *coilin* mutants *ncb-1*, *ncb-2*, and *ncb-4* were gifts from Peter J. Shaw and were isolated as described previously [28].

### Protein immunofluorescence analysis.

Isolation of nuclei from bolting seedlings and immunodetection of proteins in plant nuclei were performed as described [21,41]. Primary antibodies used included mAb Myc (1:200, Upstate), polyclonal Myc (1:200, Upstate), NRPD1b (1:100) [14], NRPD2 (1:200) [22], mAb 4G3 (anti-U2B′′) (1:100, gift from Gregory Matera), and polyclonal GFP (1:200, Invitrogen). Secondary anti-mouse-Fluorescein (Abcam), anti-rabbit-Rodamine (Jackson ImmunoResearch), anti-mouse-Rodamine (Abcam), and anti-mouse Alexa Fluor 350 (Invitrogen) were used at a dilution of 1:200. DNA was stained using Vectashield mounting medium containing DAPI (Vector Laboratories). Images were captured with the Zeiss AxioImager Z1 microscope with the Hamamatsu Orca-er camera at 100X magnification and analyzed using the Zeiss Axiovision software. Zeiss FL filter sets used in this study: Zeiss 49 (DAPI), Zeiss 38 (EGFP), and Zeiss 43 (Cy 3).

### Confocal fluorescence measurements.

Nuclei from Myc-AGO4 seedlings were examined for Myc-AGO4 and NRPD1b nuclear bodies using primary antibodies against c-Myc (1:200, Upstate) and NRPD1b (1:100). Secondary antibodies used include anti-mouse-Fluorescein (Abcam) and anti-rabbit-Rodamine (Jackson ImmunoResearch). DNA was stained using Vectashield mounting medium containing DAPI (Vector Laboratories). Confocal fluorescence measurements for AGO4, NRPD1b, and DAPI were obtained using the Zeiss LSM 510 #28 405, AR+2Hene/ 3 PMT Version 3.5 on the Zeiss Axio Imager MOT Z1 upright microscope.

### DNA FISH analysis.

DNA FISH analysis was performed essentially as described [22,42]. To combine protein immunolocalization with DNA FISH, protein immunofluorescence was first performed as described above, except nuclei slides were post-fixed in 4% paraformaldehyde after primary antibody incubation. After post-fixing, DNA FISH was performed using probes labeled with biotin-dUTP or digoxigenin (DIG)-dUTP [43]. Probes for the 45S rDNA [44], 5S rDNA [45], or the centromeric repeats [46] were generated by PCR. Biotin or DIG labeled probes were detected using FITC-avidin (1:200, Zymed), NeutrAvidin-Alexa Fluor 350 (1:200, Invitrogen), or anti-DIG-fluorescein (1:200, Roche). Vectashield mounting medium containing DAPI (Vector Laboratories) was used to stain DNA. Images were captured and analyzed as described for protein immunofluorescence, except the Apotome system was used during acquisition.

### Western blot analysis.

Protein extraction from flowers and western blot analysis were performed as described previously [21]. Equal amounts of total protein were loaded for each sample and resolved on a 10% SDS polyacrylamide gel. A monoclonal antibody to c-Myc (Upstate) was used to detect Myc-AGO4. A polyclonal antibody detecting endogenous CRY1 was used as a loading control [47].

### Southern blot analysis.

Genomic DNA extraction and southern blot analysis examining DNA methylation at *MEA-ISR* and the 5S rDNA repeats were performed as described [11,48]. Twenty μg of digested genomic DNA was loaded per sample.

### Northern blot analysis.

Total RNA extraction and northern blot analysis examining small RNAs corresponding to the 45S rDNA, 5S rDNA, and miR159 were performed as described [2,13]. Twenty μg of total RNA was loaded per sample. Probe sequences are as follows: 45S siRNA: 5′-GTCTGTTGGTGCCAAGAGGGAAAAGGGCTAAT-3′; 5S siRNA: 5′-ATGCCAAGTTTGGCCTCACGGTCT-3′; miR159: 5′-TAGAGCTCCCTTCAATCCAAA-3′.

## Supporting Information

Figure S1 Immunofluorescence Analysis of SmD3 and SmB Localization Myc-AGO4 (top panels) or NRPD1b (bottom panels) nuclear localization relative to GFP-SmD3 or GFP-SmB is shown. GFP fluorescence was used to visualize SmD3 or SmB within plant nuclei to mark the Cajal body.
(2.6 MB TIF)

Figure S2 Intensity Measurements of the Two Classes of AGO4 Nuclear Bodies (A) Fluorescence measurements of AGO4, NRPD1b, and DAPI at the different AGO4 bodies. One representative nucleus containing AGO4 colocalized with NRPD1b (left) or the Cajal body (right) used for the analysis is shown. The red arrow indicates the location in the nucleus where measurements were taken. Distance is set at zero where the highest AGO4 signal was measured. Measurements were obtained using a confocal microscope.
(B) Average intensity measurements for AGO4, NRPD1b, and DAPI at the two different AGO4 bodies. Fifteen nuclei containing AGO4/NRPD1b or AGO4/Cajal bodies were used for the averaging.
(1.5 MB TIF)

Figure S3 Localization of HYL1 Relative to AGO4, NRPD1b, and U2B′′ (A) Immunofluorescence analysis of GFP-HYL1 and Myc-AGO4 nuclear localization. GFP fluorescence was used to visualize GFP-HYL1 within the nucleus to mark the Dicing body. An antibody to Myc was used to detect Myc-AGO4.
(B) Localization analysis of GFP-HYL1 relative to NRPD1b (top panel) or U2B′′ (bottom panel) within plant nuclei. An antibody to NRPD1b or U2B′′ was used to detect endogenous NRPD1b or U2B′′ protein, respectively.
(3.0 MB TIF)

Figure S4 U2B′′ Immunostaining in *coilin* SALK Mutants Nuclei isolated from four SALK lines containing a T-DNA insertion in *COILIN* were examined for Cajal body formation by U2B′′ immunostaining. No Cajal body was observed in SALK 148589, 148630, and 010395 mutant lines. SALK 083448 is a hypomorphic allele in which a small percentage of nuclei contained a Cajal body. The remaining 2% of nuclei from wild type (Col) did not contain an observable Cajal body by U2B′′ immunostaining.
(2.8 MB TIF)

Figure S5 DNA Methylation and siRNA Levels in the *coilin* Mutants and Overexpressor (A) Southern blot analysis examining DNA methylation at the 5S rDNA repeats. Genomic DNA was digested with methyl-sensitive enzymes *Hae*III (GG^m^CC) or *Hpa*II (C^m^CGG). The triple DNA methyltransferase mutant *drm1 drm2 cmt3* is a control for the loss of DNA methylation.
(B) Southern blot analysis examining DNA methylation at *MEA-ISR*. Genomic DNA was digested with *Msp*I. The methylated DNA band is labeled “M”, and the unmethylated band is labeled “U”.
(C) Northern blot analysis examining levels of siRNAs corresponding to the 45S and 5S rDNA repeats. The microRNA miR159 was used as a loading control.
(D) Southern blot analysis examining 5S rDNA methylation levels in the *ncb-1 dcl3* double mutant. Genomic DNA was digested with *Hae*III. Two independent biological replicates of *dcl3* and *ncb-1 dcl3* were examined.
(2.7 MB TIF)

Figure S6 NRPD2 Nuclear Bodies in *Arabidopsis* Nuclei Different NRPD2 localization patterns relative to AGO4 within Myc-AGO4 nuclei are shown. The white arrow indicates an AGO4 focus that did not colocalize with NRPD2. The yellow arrow shows an NRPD2 nuclear body that did not colocalize with AGO4. Nuclei containing observable AGO4 or NRPD2 foci were examined.
(2.4 MB TIF)

Figure S7 DNaseI Controls for the DNA FISH Experiments Sample slides were treated with DNaseI (7.5 U, Roche) for 30 min at room temperature after primary antibody incubation and postfixing for DNA FISH. DNaseI treated samples did not show hybridization with the 45S rDNA, 5S rDNA, or CEN probe.
(2.1 MB TIF)

Figure S8 Localization of AGO4 or Cajal Body Relative to the NORs (A) 45S rDNA FISH combined with Myc-AGO4 immunofluorescence analysis. Nuclei containing NORs surrounded by two AGO4 foci (top panel) or three AGO4 foci (bottom panel) were observed. The remaining 80% of nuclei contained one AGO4 focus per NOR. Only nuclei containing NORs with adjacent AGO4 foci were examined.
(B) Localization of GFP-SmD3 or GFP-SmB relative to the condensed 45S rDNA loci. A polyclonal antibody to GFP was used to detect GFP-SmD3 or GFP-SmB within nuclei.
(1.6 MB TIF)

Figure S9 DRM2 and U2B′′ Localization DRM2 localization relative to the Cajal body was examined in DRM2-Myc nuclei. Nuclei containing an observable Cajal body marked by U2B′′ immunostaining were examined.
(880 KB TIF)

Table S1 Nuclei Counts for SmD3 or SmB Localization Relative to AGO4 (71 KB PDF)

Table S2 Nuclei Counts for HYL1 Localization (68 KB PDF)

Table S3 Nuclei Counts for the *coilin* Mutant (93 KB PDF)

Table S4 Nuclei Counts for RdDM Mutants (84 KB PDF)

Table S5 Nuclei Counts for NRPD1b Localization in *ago4* and *nrpd2* Mutants (94 KB PDF)

Table S6 Nuclei Counts for NRPD2 Localization (79 KB PDF)

Table S7 Nuclear Foci Counts for 45S, 5S, and CEN FISH Experiments (70 KB PDF)

Table S8 Nuclear Foci Counts for NOR2 and NOR4 (68 KB PDF)

Table S9 Nuclei Counts for DRM2-Myc Localization (80 KB PDF)
