# Supplementary material for: Dynamic Regulation of ARGONAUTE4 within Multiple Nuclear Bodies in Arabidopsis thaliana
Source: PLoS Genet. 2008 Feb 8;4(2):e27. doi: 10.1371/journal.pgen.0040027 (PMC2233678; doi:10.1371/journal.pgen.0040027)
Supplement: Supplementary file 1 [file pgen.0040027.st001.pdf]

Table S1. Nuclei counts for SmD3 or SmB localization relative to AGO4

	Colocalization	Different localization	Colocalization and different localization#	Total nuclei* (n)
SmD3 and AGO4	109 (96%)	2 (2%)	2 (2%)	113
SmB and AGO4	147 (95%)	4 (3%)	3 (2%)	154

#Nucleus contains two (or more) AGO4 foci in which one AGO4 body colocalizes with the indicated protein (SmD3 or SmB), and a second AGO4 body that is non-colocalizing

*Nuclei containing observable AGO4 nuclear foci were examined
