# Supplementary material for: Dynamic Regulation of ARGONAUTE4 within Multiple Nuclear Bodies in Arabidopsis thaliana
Source: PLoS Genet. 2008 Feb 8;4(2):e27. doi: 10.1371/journal.pgen.0040027 (PMC2233678; doi:10.1371/journal.pgen.0040027)
Supplement: Supplementary file 2 [file pgen.0040027.st002.pdf]

Table S2. Nuclei counts for HYL1 localization

	Colocalization	Different localization	Total nuclei* (n)
HYL1 and AGO4	0 (0%)	99 (100%)	99
HYL1 and NRPD1B	0 (0%)	59 (100%)	59
HYL1 and U2B”	0 (0%)	80 (100%)	80

*Nuclei containing both HYL1 and AGO4/NRPD1b/U2B” nuclear foci were examined
