# Supplementary material for: Dynamic Regulation of ARGONAUTE4 within Multiple Nuclear Bodies in Arabidopsis thaliana
Source: PLoS Genet. 2008 Feb 8;4(2):e27. doi: 10.1371/journal.pgen.0040027 (PMC2233678; doi:10.1371/journal.pgen.0040027)
Supplement: Supplementary file 3 [file pgen.0040027.st003.pdf]

Table S3. Nuclei counts for the *coilin* mutant

	Co-localization	Different localization	Colocalization and different localization*	No AGO4 body	Total nuclei (n)
Wild type:					
U2B” and AGO4	118 (87%)	4 (3%)	10 (7%)	4 (3%)	136
NRPD1b and AGO4	4 (3%)	121 (88%)	9 (7%)	3 (2%)	137
coilin mutant:					
U2B” and AGO4	0 (0%)	28 (13%)	0 (0%)	180 (87%)	208
NRPD1b and AGO4	24 (14%)	0 (0%)	0 (0%)	152 (86%)	176

*Nucleus contains two (or more) AGO4 foci in which one AGO4 body colocalizes with the indicated protein (U2B” or NRPD1b), and a second AGO4 body that is non-colocalizing
