# Supplementary material for: Dynamic Regulation of ARGONAUTE4 within Multiple Nuclear Bodies in Arabidopsis thaliana
Source: PLoS Genet. 2008 Feb 8;4(2):e27. doi: 10.1371/journal.pgen.0040027 (PMC2233678; doi:10.1371/journal.pgen.0040027)
Supplement: Supplementary file 4 [file pgen.0040027.st004.pdf]

Table S4. Nuclei counts for RdDM mutants

	Co-localization	Different localization	Colocalization and different localization*	No AGO4 body/ staining	Total nuclei (n)
Wild type:					
NRPD1b and AGO4	3 (2%)	171 (86%)	18 (9%)	6 (3%)	198
dcl3 mutant:					
NRPD1b and AGO4	8 (4%)	27 (13%)	18 (8%)	163 (75%)	216
Wild type:					
NRPD1b and AGO4	20 (14%)	112 (78%)	7 (5%)	5 (3%)	144
U2B” and AGO4	71 (84%)	9 (11%)	3 (3%)	2 (2%)	85
nprd1b mutant:					
NRPD1b and AGO4	0 (0%)	173 (94%)	0 (0%)	11 (6%)	184
U2B” and AGO4	120 (94%)	0 (0%)	0 (0%)	7 (6%)	127
drm2 mutant:					
NRPD1b and AGO4	0 (0%)	119 (85%)	18 (13%)	3 (2%)	140
U2B” and AGO4	116 (88%)	0 (0%)	12 (9%)	4 (3%)	132

*Nucleus contains two (or more) AGO4 foci in which one AGO4 body colocalizes with the indicated protein (NRPD1b or U2B”), and a second AGO4 body that is non-colocalizing
