# Supplementary material for: Dynamic Regulation of ARGONAUTE4 within Multiple Nuclear Bodies in Arabidopsis thaliana
Source: PLoS Genet. 2008 Feb 8;4(2):e27. doi: 10.1371/journal.pgen.0040027 (PMC2233678; doi:10.1371/journal.pgen.0040027)
Supplement: Supplementary file 5 [file pgen.0040027.st005.pdf]

Table S5. Nuclei counts for NRPD1b localization in *ago4* and *nrpd2* mutants

	Both NRPD1b and U2B" present	Only U2B" present	Total nuclei (n)
Ler:			
NPRD1b and U2B"	18 (10%)	167 (90%)	185
ago4 mutant:			
NPRD1b and U2B"	8 (4%)	194 (96%)	202
Col:			
NRPD1b and U2B"	16 (7%)	219 (93%)	235
nrpd2a nrpd2b mutant:			
NRPD1b and U2B"	0 (0%)	243 (100%)	243
