# Supplementary material for: Dynamic Regulation of ARGONAUTE4 within Multiple Nuclear Bodies in Arabidopsis thaliana
Source: PLoS Genet. 2008 Feb 8;4(2):e27. doi: 10.1371/journal.pgen.0040027 (PMC2233678; doi:10.1371/journal.pgen.0040027)
Supplement: Supplementary file 6 [file pgen.0040027.st006.pdf]

Table S6. Nuclei counts for NRPD2 localization

	Co-localization	Different localization	U2B” present only	Total nuclei (n)
NPRD2 and U2B” in:				
Col	0 (0%)	17 (8%)	201 (92%)	218
nprd1a	0 (0%)	11 (6%)	164 (94%)	175
nrpd1b	0 (0%)	0 (0%)	177 (100%)	177
Ler	0 (0%)	20 (9%)	196 (91%)	216
ago4	0 (0%)	13 (7%)	172 (93%)	185
