# Supplementary material for: Dynamic Regulation of ARGONAUTE4 within Multiple Nuclear Bodies in Arabidopsis thaliana
Source: PLoS Genet. 2008 Feb 8;4(2):e27. doi: 10.1371/journal.pgen.0040027 (PMC2233678; doi:10.1371/journal.pgen.0040027)
Supplement: Supplementary file 7 [file pgen.0040027.st007.pdf]

Table S7. Nuclear foci counts for 45S, 5S, and CEN FISH experiments

	Not adjacent	Adjacent	Overlapping	Total nuclear foci (n)
45S rDNA with AGO4	91 (45%)	100 (50%)	10 (5%)	201
45S rDNA with NRPD1b	0 (0%)	85 (89%)	11 (11%)	96
45S rDNA with SmD3	149 (93%)	8 (5%)	3 (2%)	160
45S rDNA with SmB	137 (89%)	11 (7%)	6 (4%)	154
5S rDNA with AGO4	170 (92%)	12 (7%)	2 (1%)	184
5S rDNA with NRPD1b	65 (90%)	6 (8%)	1 (2%)	72
CEN with AGO4	134 (91%)	8 (6%)	5 (3%)	147
CEN with NRPD1b	64 (89%)	5 (7%)	3 (4%)	72
