# Supplementary material for: Dynamic Regulation of ARGONAUTE4 within Multiple Nuclear Bodies in Arabidopsis thaliana
Source: PLoS Genet. 2008 Feb 8;4(2):e27. doi: 10.1371/journal.pgen.0040027 (PMC2233678; doi:10.1371/journal.pgen.0040027)
Supplement: Supplementary file 8 [file pgen.0040027.st008.pdf]

Table S8. Nuclear foci counts for NOR2 and NOR4

	NOR2 adjacent[1]	NOR4 adjacent[2]	Total nuclear foci (n)
AGO4	27 (60%)	18 (40%)	45*
NRPD1b	25 (64%)	14 (36%)	39

[1]NOR2 is counted as a 45S signal without an adjacent 5S rDNA signal
[2]NOR4 is counted as a 45S signal with an adjacent 5S rDNA signal
*only AGO4 nuclear foci adjacent to a NOR were counted
