# Supplementary material for: Dynamic Regulation of ARGONAUTE4 within Multiple Nuclear Bodies in Arabidopsis thaliana
Source: PLoS Genet. 2008 Feb 8;4(2):e27. doi: 10.1371/journal.pgen.0040027 (PMC2233678; doi:10.1371/journal.pgen.0040027)
Supplement: Supplementary file 9 [file pgen.0040027.st009.pdf]

Table S9. Nuclei counts for DRM2-Myc localization

	Co-localization	NRPD1b/ NRPD2 body only	Diffuse DRM2 staining only, no nuclear foci	Total nuclei (n)
NRPD1b and DRM2 in:				
Wild type	10 (9%)	1 (1%)	102 (90%)	113
ago4 mutant	3 (2%)	12 (9%)	120 (89%)	135
NRPD2 and DRM2	12 (5%)	9 (3%)	239 (92%)	260
